# Genetic variants and down-regulation of *CACNA1H* in pheochromocytoma

**DOI:** 10.1530/ERC-23-0061

**Published:** 2024-07-08

**Authors:** Fredrika Svahn, Karolina Solhusløkk Höse, Adam Stenman, Yaxuan Liu, Jan Calissendorff, Emma Tham, Ákos Végvári, Roman A Zubarev, Na Wang, Reju Korah, Tobias Carling, Jan Zedenius, Robert Bränström, C Christofer Juhlin, Catharina Larsson

**Affiliations:** 1 Department of Oncology-Pathology, Karolinska Institutet, Stockholm, Sweden; 2 Department of Molecular Medicine and Surgery, Karolinska Institutet, Stockholm, Sweden; 3 Department of Breast, Endocrine Tumors and Sarcoma, Karolinska University Hospital, Stockholm, Sweden; 4 Department of Breast Surgery, Obstetrics and Gynecology Hospital of Fudan University, Shanghai, China; 5 Department of Clinical Genetics, Karolinska University Hospital, Stockholm, Sweden; 6 Division of Chemistry I, Department of Medical Biochemistry and Biophysics, Karolinska Institutet, Stockholm, Sweden; 7 Department of Medicine Huddinge, Karolinska Institutet, Huddinge, Sweden; 8 Yale Endocrine Neoplasia Laboratory, Department of Surgery, Yale School of Medicine, New Haven, Connecticut, USA; 9 Carling Adrenal Center, Tampa, Florida, USA; 10 Department of Clinical Pathology and Cancer Diagnostics, Karolinska University Hospital Stockholm, Sweden

**Keywords:** CACNA1H, calcium channel, pheochromocytoma, paraganglioma

## Abstract

Pheochromocytoma (PCC) and abdominal paraganglioma (aPGL) (together abbreviated PPGL) frequently present with an underlying genetic event in a PPGL driver gene, and additional susceptibility genes are anticipated. Here, we re-analyzed whole-exome sequencing data for PCC patients and identified two patients with rare missense variants in the calcium voltage-gated channel subunit 1H gene (*CACNA1H*). *CACNA1H* variants were also found in the clinical setting in PCC patients using targeted sequencing and from analysis of The Cancer Genome Atlas database. In total, *CACNA1H* variants were found in six PCC cases. Three of these were constitutional, and two are known to have functional consequences on hormone production and gene expression in primary aldosteronism and aldosterone-producing adrenocortical adenoma. In general, PPGL exhibited reduced *CACNA1H* mRNA expression as compared to normal adrenal. Immunohistochemistry showed strong CACNA1H (Ca_V_3.2) staining in adrenal medulla while PPGL typically had weak or negative staining. Reduced *CACNA1H* gene expression was especially pronounced in PCC compared to aPGL and in PPGL with cluster 2 kinase signaling phenotype. Furthermore, *CACNA1H* levels correlated with *HIF1A* and *HIF2A*. Moreover, TCGA data revealed a correlation between *CACNA1H* methylation density and gene expression. Expression of rCacna1h in PC12 cells induced differential protein expression profiles, determined by mass spectrometry, as well as a shift in the membrane potential where maximum calcium currents were observed, as determined by electrophysiology. The findings suggest the involvement of *CACNA1H/*Ca_V_3.2 in pheochromocytoma development and establish a potential link between the etiology of adrenomedullary and adrenocortical tumor development.

## Introduction

Pheochromocytoma (PCC) is a neuroendocrine tumor that arises from chromaffin cells in the adrenal medulla, and abdominal paraganglioma (aPGL) exhibits a highly related cellular origin (Mete *et al.* 2022). Based on their similarities, PCC and aPGL are frequently grouped together as PPGL. Affected patients commonly exhibit symptoms due to increased catecholamine production. PPGLs have a prominent genetic background, as up to 40% of cases carry a constitutional mutation in one of the known susceptibility genes (Mete *et al.* 2022, Buffet *et al.* 2020, Juhlin *et al.* 2015, Hadrava Vanova *et al.* 2022), which partly overlap the somatic mutation spectra in PPGL (Liu *et al.* 2014, Welander *et al.* 2018, Mete *et al.* 2022).

Based on gene expression profiling, three molecular subgroups of PPGL have been identified (Fishbein *et al.* 2017, Juhlin 2021). Cluster 1 tumors demonstrate a pseudohypoxia expression profile, exhibit a hypermethylation phenotype, are more prone to metastasize, and are more likely of extra-adrenal origin (Fishbein *et al.* 2017, Jochmanova & Pacak 2018). Cluster 2 tumors (Fishbein *et al.* 2017, Jochmanova & Pacak 2018) display activation of kinase signaling pathways, are typically of adrenal origin, and usually show a non-metastatic course with good patient outcomes, with the exception of tumors displaying *ATRX* mutations (Jochmanova & Pacak 2018). Furthermore, a Wnt-altered subtype has been recognized (Fishbein *et al.* 2017).

**Figure 1:**
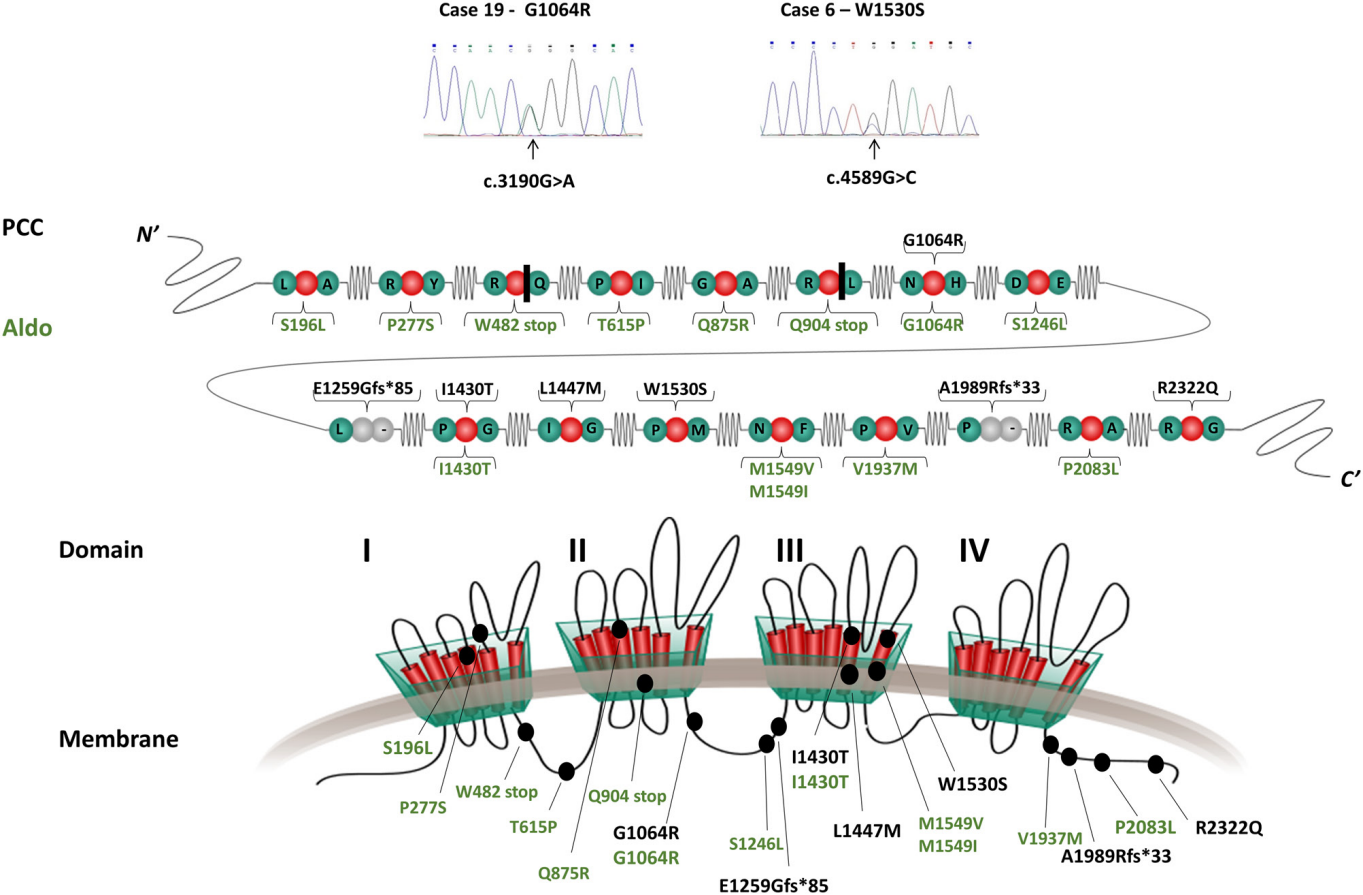
The CACNA1H protein and detected variants. Top: sequencing chromatograms from Sanger sequencing showing *CACNA1H* variants in tumor tissue from three PCC cases with the same variants identified in constitutional tissue (Table 1). Middle: schematic illustration of protein alterations predicted from variants detected in pheochromocytoma (PCC, black) and previously reported in primary hyperaldosteronism/aldosterone-producing adrenocortical adenoma (Aldo, green). Bottom: location of PCC and Aldo variants (black ovals) in the CACNA1H protein complex consisting of four domains (I−IV) each with six individual segments (red bars).

The alpha 1 subunit of the family of T-type low voltage-operated calcium channels (VOCCs) includes three isoforms encoded by the genes *CACNA1G* (Ca_V_3.1), *CACNA1H* (Ca_V_3.2), and *CACNA1I* (Ca_V_3.3) (Weiss & Zamponi 2020). The alpha 1 subunit forms four complexes each with six segments (Fig. 1), of which segments 5 and 6 will form the pore needed for calcium influx (Scholl *et al.* 2015). These channels are involved in neural excitability and play a role in hormone release in neuroendocrine organs (Weiss & Zamponi 2020). VOCCs have important functions in physiological processes, and several channelopathies have been linked to mutations in the encoding genes (Weiss & Zamponi 2020). Constitutional *CACNA1H* mutations are an underlying cause of familial forms of primary hyperaldosteronism, characterized by hypertension at a young age due to increased calcium influx in adrenocortical cells of the zona glomerulosa (Scholl *et al.* 2015). Subsequently, somatic *CACNA1H* mutations have been found to underlie the development of subsets of sporadic aldosterone-producing adrenocortical adenomas (Nanba *et al.* 2020). In PC12 rat pheochromocytoma cells, hypoxia increases the expression of *CACNA1H* / Ca_V_3.2 and its function, with an increase in Ca_V_3.2-mediated calcium influx (Sellak *et al.* 2014). Furthermore, hypoxia-inducible factor 1 alpha (HIF1A) binds to a region of the *CACNA1H* promoter (Sellak *et al.* 2014), which is of particular interest for PPGL. In addition, cluster 1 tumors have a molecular background resulting in HIF activation, and* HIF2A* (*EPAS1*) is one of the known tumorigenic drivers in PPGL.

To further clarify the molecular genetic background of PPGL, we analyzed previously generated unpublished constitutional data from a next-generation sequencing study (Juhlin *et al.* 2015). Based on the identification of genetic variants in the VOCC *CACNA1H*, investigations were performed in a larger case series concerning gene and protein expression and their relation to clinical presentation and molecular phenotypes, as well as effects on protein expression profiles and calcium currents.

## Material and methods

### Discovery PCC cohort

Fifteen PCC cases from the Karolinska University Hospital were previously analyzed by whole-exome sequencing (WES). In a previous report, somatic mutations were found by comparing PCC tumors with matched normal tissue, and the data were mapped to the human reference genome (Juhlin *et al.* 2015).

### The Karolinska PPGL cohort

Tumor tissue samples from 97 primary PPGL cases (85 PCC and 12 aPGL) operated on at Karolinska University Hospital were used in the study. The full clinical and genetic information is detailed in Supplementary Table 1, see section on supplementary materials given at the end of this article. Detailed data for the pheochromocytoma of the adrenal gland scaled score (PASS) have been published for the majority of cases (Stenman *et al.* 2019). All tumors were previously investigated for mutations in PPGL-related genes (Supplementary Table 1), and a subset of the tumors were subjected to WES analysis (Welander *et al.* 2014, Juhlin *et al.* 2015, Stenman *et al.* 2016*a*). Affymetrix-based mRNA expression profiles were previously generated for the majority of tumors (Stenman *et al.* 2016*b*, 2019). Based on the mutation status and/or mRNA expression profile, tumors were assigned as cluster 1 (*SDHx*, *VHL*, *EPAS1,* and *EGLN1*) or cluster 2 (*RET*, *NF1*, *TMEM127*, *MAX*, and *HRAS*).

Fresh frozen tumor tissue samples were collected from the local Biobank for all 97 PPGL. For 29 of the cases, formalin-fixed paraffin-embedded (FFPE) PPGL slides were also obtained for immunohistochemistry. Fresh frozen non-tumorous adrenal tissue samples were used as references for qRT-PCR. FFPE tissue sections from de-identified normal testis, normal pancreas, pancreatic neuroendocrine tumor (Pan-NET), and normal adrenal gland (from patients operated on for other metastatic lesions to the adrenal) were included as immunohistochemical controls.

### The TCGA cohort

For the TCGA cohort of PPGL (Fishbein *et al.* 2017), the Firehose Legacy datasets used were downloaded from https://www.cbioportal.org/ in August 2020. Only primary tumors with a histological diagnosis of PCC or aPGL were selected. Head and neck PGL were excluded, and only one sample per patient was enrolled. The resulting cohort consisted of 177 samples (148 PCC and 29 aPGL) from 100 females and 77 males, with a median age of 46 years at diagnosis (range 19–83 years). Fifteen patients had recurrent or metastatic disease. Methylation density values for *CACNA1H* were downloaded from UCSC Xena Functional Genomics Explorer https://xenabrowser.net/ in September 2020. Data for mRNA expression clusters 1 or 2, and DNA methylator cluster (hyper-methylated M1 or intermediate/low-methylated non-M1) were obtained from Fishbein *et al.* (2017).

The following data were obtained and analyzed: *CACNA1H* variants and mutation status of known PPGL susceptibility genes for the three cases with a *CACNA1H* variant; mRNA expression profiles; *CACNA1H* mRNA expression; *CACNA1H* DNA methylation; *CACNA1H* copy number status; *HIF1A* mRNA expression; *EPAS1* (*HIF2A*) mRNA expression; mRNA cluster M1/non-M1 phenotype; and clinical data for patient sex, tumor type, tumor weight, metastasis, and absolute outcome.

### Analysis of next-generation sequencing data

WES data were used from the previously published discovery cohort (Juhlin *et al.* 2015). In this study, candidate PPGL genes were searched for, this time focusing on variants present in both tumor and matched constitutional tissue but absent in the human reference genome and absent or rare in gnomAD. An additional case (E2) was detected by clinical routine sequencing via the Clinical Genetics Department at the Karolinska University Hospital. For case E2, a tissue sample from normal myocardium was obtained at autopsy with consent from the nearest of kin; however, the tumor was not available. WES analysis was performed with analysis of an established gene panel for endocrine tumors (Supplementary Table 3) as described in Supplementary Document (Lindstrand *et al.* 2019, Muth *et al.* 2019). Detected variants were assessed for predictions of deleterious variants and allele frequencies from gnomAD, as detailed in Supplementary Document.

### Sanger sequencing

Ninety-five PPGL were screened for variants in “hotspot” areas of exons 16, 17, 22, 25, and 34 of the *CACNA1H* gene using primers detailed in Supplementary Table 4 and experimental procedures described in Supplementary Document.

### Quantitative real-time polymerase chain reaction (qRT-PCR)

*CACNA1H* mRNA expression was successfully analyzed by qRT-PCR in 83 PPGL and eight normal adrenal tissue samples using assays for *CACNA1H* (Hs00234934, Thermo Fisher) and the housekeeping gene *B2M* (Hs99999907, Thermo Fisher), following experimental procedures described in Supplementary Document.

### Immunohistochemistry

CACNA1H protein expression was investigated by immunohistochemistry in 29 PPGL cases (20 PCC and nine PGL; Supplementary Table 5) as described in detail in Supplemental Document. When available, normal adrenal medulla and/or cortex on the same slide was used for comparison. Normal testis, normal pancreas, a Pan-NET, and normal adrenal were analyzed in parallel as controls. Slides were incubated with primary antibody against the T-type Ca^++^ CP α1H (G-10, sc-377510, Santa Cruz Biotechnology) at a dilution 1:400. The results were evaluated by a pathologist (CCJ) blinded to clinical and genetic data, and cytoplasmatic immunoreactivity was graded as: minimal or no staining (0); mixed staining pattern with negatively and positively stained cells (±); weak staining intensity (+); or strong staining intensity (++).

### Cell culture, plasmids, and transfection

The established rat adrenal pheochromocytoma cell line PC12 was used for overexpression of rat rCacna1h and analyzed as detailed in Supplementary Document.

### Mass spectrometry, data analyses, and protein ontology analyses

Mass spectrometry analysis of PC12 cells transfected with wild-type rCacna1h plasmid and control vector plasmid was performed at the Proteomics Biomedicum core facility at Karolinska Institutet, applying previously reported procedures (Shafi *et al.* 2023) with modifications as detailed in Supplementary Document.

### Electrophysiology

For electrophysiological experiments, the analyses largely followed previously published experimental procedures (Lu *et al.* 2010) with modifications as described in Supplementary Document. PC12 cells transfected with wild-type rCacna1h plasmid and control vector plasmid were incubated in petri dishes at 37˚C and 5% CO_2_ overnight, and VOCCs activity was recorded using the patch-clamp technique (Hamill *et al.* 1981).

### Statistical analyses and illustrations

IBM SPSS Statistics Version 25 and 26 were used for statistical analyses and graphic work, as described in Supplementary Document.

## Results

### Identification of *CACNA1H* variants

Previously generated and unpublished WES data of constitutional DNA for the 15 PCC cases in the discovery cohort were investigated and compared to the public reference genome (Juhlin *et al.* 2015). This identified two cases (6 and 19) that carried *CACNA1H* missense variants, W1530S and G1064R, respectively (Table 1, Supplementary Table 2). Since constitutional *CACNA1H* variants have previously been associated with primary hyperaldosteronism (Scholl *et al.* 2015), *CACNA1H* was selected for further analysis. One additional constitutional *CACNA1H* variant was subsequently found in a PCC patient from WES with analysis of an endocrine tumor gene panel (Supplementary Table 3) in the clinical routine setting during the study period. An indel frameshift A1989Rfs*33 was detected in constitutional tissue from this case E2 (Fig. 1, Table 1). Investigation of the TCGA dataset of 177 PPGL tumors identified three additional *CACNA1H* variants, two of which were missense variants (I1430T and L1447M) and one frameshift deletion (E1259Gfs*85) (Table 1).

**Table 1:** Details of *CACNA1H* variants detected in human PCC cases in the Karolinska cohort and the TCGA database.

Case number	Type of variant	Exon no.	Genetic alteration	Protein effect	Detected in		Location in CACNA1H^a^		Reported in/functional consequences^b^
					Normal	Tumor			
**Karolinska cohort**									
6	Missense	25	c.4589G>C	W1530S	Blood	Prim tumor	Membrane	Repeat III-S6 (pore-forming)	–
19	Missense	16	c.3190G>A	G1064R	Blood	Prim tumor	Cytoplasm	Between repeat II and III	Primary hyperaldosteronism/aldosterone production
E2	FS del	34	c.5965_5987del	A1989Rfs*33	Myocardium	–	Cytoplasm	After repeat IV	–
**TCGA database**									
WB-A81D	Missense	22	c.4289T>C	I1430T	Not reported	Prim tumor	Membrane	Repeat III-S5 (pore-forming)	Aldosterone-producing adrenocortical adenoma (S)/aldosterone production
QR-A70U	Missense	22	c.4339T>A	L1447M	Not reported	Prim tumor	Membrane	Repeat III-S5 (pore-forming)	–
S7-A7WP	FS del	18	c.3776del	E1259Gfs*85	Not reported	Prim tumor	Cytoplasm	Between repeat II and III	–

TCGA database and Fishbein *et al.* 2017 by comparing tumor and normal tissue. Reference sequence NM_021098.3. ^a^According to Uniprot.org; ^b^ Roomp *et al.* 2016, Nanba *et al.* 2020.

FS del, frameshift deletion; Prim tumor, primary tumor; S, somatics.

The Karolinska cohort of 95 PPGL tumors was successfully screened for *CACNA1H* variants by Sanger sequencing of five focal areas of exons 16, 17, 22, 25, and 34 based on the localization of the *CACNA1H* variants (Fig. 1) found previously. The investigation verified the presence of the tumor DNA of the *CACNA1H* variants initially found by WES in cases 6 and 19 but did not reveal additional deleterious variants.

In total, six potentially damaging *CACNA1H* variants were found in the Karolinska cohort and in the TCGA database (Table 1). Two variants (W1530S and G1064R) have been reported in gnomAD at frequencies less than 0.0001, while the other four variants (A1989Rfs*33, I1430T, L1447M, and E1259Gfs*85) are not known in gnomAD, suggesting they are very uncommon in the general population (Table 2). *In silico* predictions and reported frequencies are summarized in Table 2, which revealed that the missense variants were predicted as pathogenic by two or more prediction tools. Based on WES data from tumor and/or constitutional DNA, mutations in known PPGL genes were not revealed in any of the six cases (Table 3).

**Table 2:** *In silico* predictions and reported frequencies of detected *CACNA1H* variants.

	W1530S	G1064R	A1989Rfs*33	I1430T	L1447M	E1259Gfs*85
Cohort	Karolinska	Karolinska	Karolinska	TCGA	TCGA	TCGA
Case no.	6	19	E2	WB-A81D	QR-A70U	S7-A7WP
DNA alteration	c.4589G>C	c.3190G>A	c.5965_5987del	c.4289T>C	c.4339T>A	c.3776del
Conserved amino acid	Highly	Highly	–	Highly	Highly	–
***In silico***** predictions**						
Mutation Taster (v2021)	Deleterious	Deleterious	Deleterious	Deleterious	Benign	Deleterious
Tree vote (del/benign)	75/25	69/31	–	86/14	27/73	–
PolyPhen2	Probably damaging	Probably damaging	–	Probably damaging	Probably damaging	–
Score	0.998	1.000	–	0.998	0.999	–
CADD (v1.6) phred score	29.4	28.9	–	25.1	21.8	–
SIFT (v6.2.0)	Deleterious	Deleterious	–	Deleterious	Deleterious	–
Score	0.00	0.00	–	0.00	0.00	–
Align GVGD (v2007)	Benign	Likely benign	–	Damaging	Benign	–
Class	C0	C15	–	C65	C0	–
Grantham distance	Large	Moderate	–	Moderate	Small	–
**gnomAD**						
Variant ID	16-1261968-G-C	16-1258048-G-A	Not reported	Not reported	Not reported	Not reported
Frequency all	0.0016%	0.0051%	None	None	None	None
EUR allele frequency	0.0035%	0.0075%	None	None	None	None
EUR carrier frequency	1 in 14,500	1 in 7000	None	None	None	None

Reference sequence NM_021098.3.

FS del, frameshift deletion.

**Table 3:** Clinical details of human PCC cases with detected *CACNA1H* variants.

Panel number	Age (years)	Sex (M/F)	Diagnosis	Size (mm)	Malignant behavior	Follow-up		Other tumors	Other diseases and features	Wild-type PPGL genes	
						Time	Outcome			Constitutional	Tumor
**Karolinska cohort**											
6	44	F	PCC	20	No	16.7 y	Alive	Breast cancer	Anosmia, toxic MNG	By WES^a^	By WES^a^
19	37	M	PCC	100	No	27 y	Alive	Seminoma neurofibromas	Vertigo	By WES^a^	By WES^a^
E2	51	M	PCC	45	No	0 y	Dead	–	––	By WES-panel^b^	–
**TCGA database**											
TCGA-WB-A81D	82	F	PCC	75	No	4 y	Alive	–	–	By WES^c^	By WES^c^
TCGA-QR-A70U	46	F	PCC	57	No	4 y	Alive	–	–	By WES^c^	By WES^c^
TCGA-S7-A7WP	25	F	PCC	50	No	2 y	Alive	–	–	By WES^c^	By WES^c^

Age, age at diagnosis; F, female; M, male; MNG, multinodular goiter; −, not reported; PCC, pheochromocytoma;WES, whole-exome sequencing; y, years.

^a^Juhlin *et al.* 2015; ^b^Gene panel detailed in the Supplementary Table 3; ^c^Fishbein *et al*. 2017.

All *CACNA1H* variants are summarized in Tables 1, 2, and3 and schematically illustrated in Fig. 1. All four missense variants are located in evolutionarily conserved amino acids (Supplementary Fig. 1). The missense variants W1530S, I1430T, and L1447M are located in the membrane, affecting the pore-forming segments 5 or 6 in repeat III, while G1064R is located in the cytoplasm (Fig. 1, Table 1). The two frameshift variants A1989Rfs*33 and E1259Gfs*85 occur at amino acids located in the cytoplasm, resulting in truncation/loss of amino acids after repeat IV and between repeats III/IV, respectively (Fig. 1). Two variants, G1064R and I1430T, have also been reported constitutionally in a family with primary hyperaldosteronism and as a recurrent somatic event in aldosterone-producing aldosteronoma, respectively (Roomp *et al.* 2016, Nanba *et al.* 2020).

Clinical details are summarized in Table 3 and described for cases 6, 19, and E2 in Supplementary Document. None of the three cases from Karolinska with a *CACNA1H* variant showed signs of primary hyperaldosteronism at the time of diagnosis, neither from clinical chemistry analyses nor at subsequent histopathology work-up.

### Expression of CACNA1H / Ca_V_3.2 is frequently suppressed in PPGL as compared to adrenal medulla

**Figure 2:**
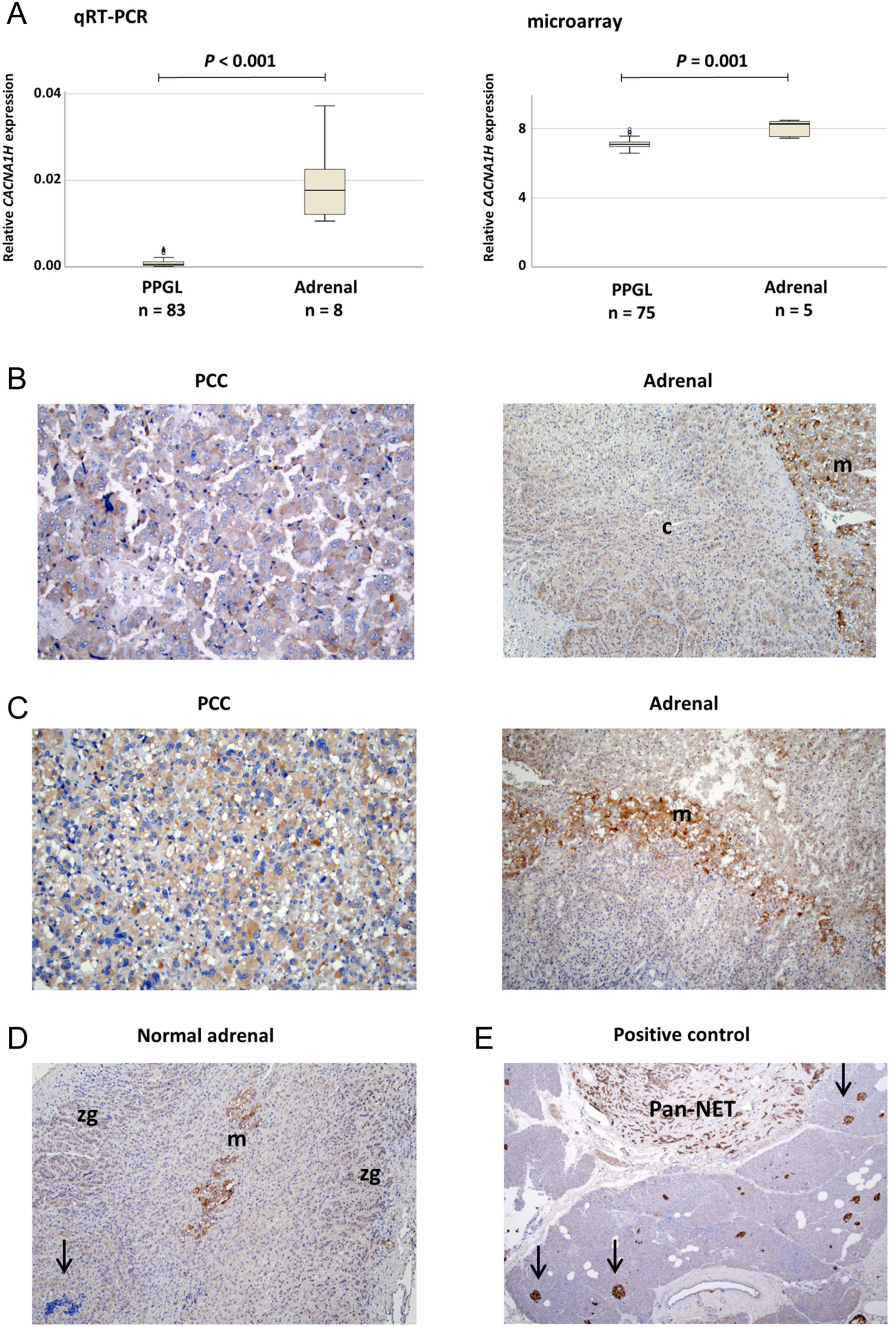
Comparison of CACNA1H expression in PPGL and adrenal tissues. (A) Relative mRNA expression of *CACNA1H* determined by qRT-PCR (left) and from microarray data (right) in pheochromocytoma/abdominal paraganglioma (PPGL) from the Karolinska cohort and in adrenal references. (B) Immunohistochemical analysis of CACNA1H protein expression in case 66. The PCC shows weak staining (+) at ×200 magnification. Non-tumorous adrenal tissue from the same case shows strong staining (++) in medulla (m) and weak staining (+) in cortex (c) at ×100. (C) Case 71 showing weak (+) CACNA1H expression in PCC tissue (at ×200) and strong staining (++) in medulla (at ×100). (D) Positive CACNA1H staining in adrenal tissue from a patient with non-PPGL disease at ×100 magnification. Strong expression (++) is noted in the medulla (m), while the zona glomerulosa of the cortex (zg) has weak expression (+). Negative lymphocytes are indicated (arrow). (E) Positive control showing strong CACNA1H expression (++) in a pancreatic neuroendocrine tumor (Pan-NET) and negative staining (−) in exocrine pancreas negative control at ×40 magnification. Arrows indicate positively stained Langerhans islets.

Quantification of *CACNA1H* mRNA expression in the Karolinska cohort showed that the tumor samples had lower expression compared to the adrenal references (*P* < 0.001, Mann–Whitney *U*-test, Fig. 2A). This finding also confirmed using microarray-based expressional data from a largely overlapping cohort, showing lower *CACNA1H* expression in PPGL compared to adrenal samples (*P* = 0.001, Mann–Whitney *U*-test, Fig. 2A).

CACNA1H/Ca_V_3.2 protein expression was subsequently examined by immunohistochemistry in 29 PPGL cases and reference tissues. Examples of control tissues are shown for pan-NET (positive) and pancreas (negative) in Fig. 2E. In a subset of PPGL, the presence of adrenal medulla (12 samples) and adrenal cortex (18 samples) on the same slides permitted direct comparison of the staining pattern with tumor tissue (Fig. 2; Supplementary Table 5). Strong cytoplasmic staining (++) was found in the adrenal medulla for all 12 PPGL samples, while the adrenal cortex typically showed weak staining (+) (Fig. 2B and C). A similar staining pattern was observed in normal adrenal tissue from non-PPGL disease, with strong staining of the medulla together with weak staining of the cortex, including both zona glomerulosa and zona reticularis (Fig. 2D). Among the 29 PPGL, 15 samples had weak staining (+), seven showed varied staining with a mixture of negatively and positively stained cells (±), including the two cases with missense *CACNA1H* variants (Supplementary Fig. 2), and seven cases showed strong staining (++) in the tumor tissue.

### *CACNA1H* methylation levels correlate with *CACNA1H* expression

**Figure 3:**
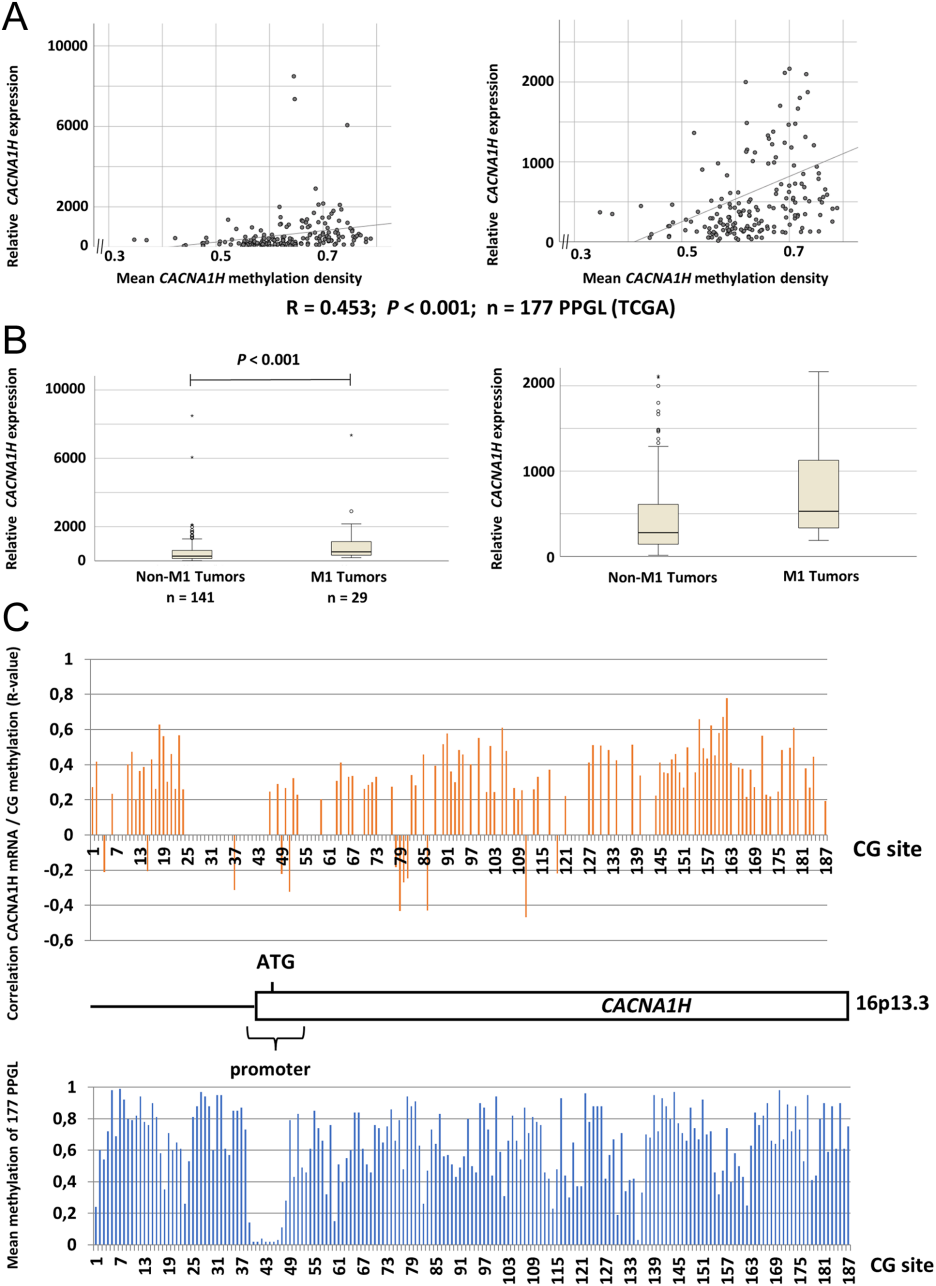
Comparison of *CACNA1H* gene expression, *CACNA1H* methylation density, and global hypermethylation phenotype using TCGA data. (A) Scatter plots showing the correlation between the mean methylation density of 187 CG sites covering the *CACNA1H* locus and *CACNA1H* mRNA expression. An enlargement, without outliers with very high expression, is shown to the right. (B) Comparison of *CACNA1H* mRNA expression levels between PPGL M1 tumors (global hypermethylated phenotype) compared to non-M1 tumors (intermediate and low methylation). Enlargement without outliers to the right. (C) Schematic illustration of the *CACNA1H* gene locus with indication of promoter region and ATG site according to Ensembl (https://www.ensembl.org). The graph above illustrates *R*-values for significant correlations with *P*-value < 0.01 between *CACNA1H* methylation and mRNA expression at the 187 CG sites. Below is shown mean methylation for 177 PPGL at the individual CG sites.

A possible correlation between *CACNA1H* methylation density and gene expression levels was investigated using the TCGA dataset (Fig. 3). The mean methylation density across the *CACNA1H* gene locus was moderately correlated with *CACNA1H* gene expression (*R* = 0.453; *P* < 0.001, Fig. 3A). With regard to the individual CG sites, highly variable methylation density was observed, determined as a mean for each of the 177 PPGLs included (range 0.02–0.99; Fig. 3C; Supplementary Table 6), with the lowest methylation density observed within the promoter region. For 113 out of 187 CG sites, there was a significant correlation between *CACNA1H* mRNA expression and methylation density (*P* < 0.01). In total, 43 of these CG sites showed a positive correlation with *R*-values above 0.4, and three sites showed an inverse correlation with *R*-values below −0.4 (Supplementary Table 6).

We also compared *CACNA1H* expression between PPGL in different global methylation clusters using data obtained from Fishbein *et al.* (2017). This revealed that the M1 hypermethylated tumor group (*n* = 29) was associated with higher *CACNA1H* mRNA levels compared to the non-M1 tumors (*n* = 141) (*P* = 0.001; Mann–Whitney *U*-test; Fig. 3B).

### Low *CACNA1H* mRNA levels are associated with PCC and cluster 2 phenotype

**Figure 4:**
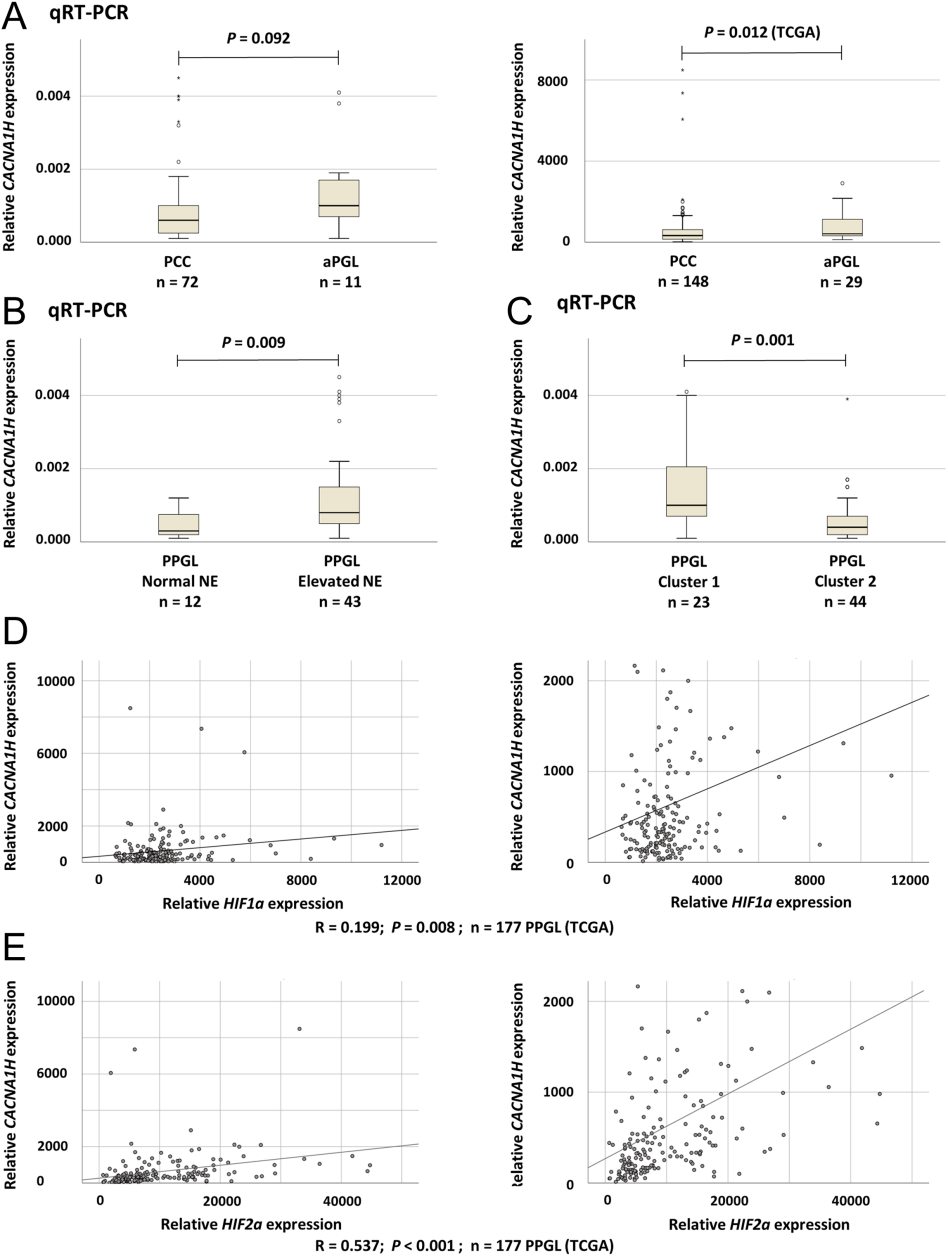
Comparison of *CACNA1H* mRNA expression with tumor type, norepinephrine (NE) secretion, and cluster 1 or 2 phenotype. (A) Relative *CACNA1H* mRNA expression in PCC and aPGL determined by qRT-PCR in the Karolinska cohort (left) and based on data from the TCGA database (right). (B) *CACNA1H* mRNA expression in PPGL of the Karolinska cohort with elevated or normal levels of NE. (C) *CACNA1H* mRNA expression in PPGL of the Karolinska cohort with cluster 1 (pseudo-hypoxia) or cluster 2 (kinase signaling) expression phenotype. (D) Correlation analysis between *CACNA1H* and *HIF1A* mRNA expression based on data from the TCGA database. An enlargement, without outliers with very high expression, is shown to the right. (E) Correlation analysis between *CACNA1H* and *EPAS1* (*HIF2A*) mRNA expression based on data from the TCGA database. An enlargement, without outliers with very high expression, is shown to the right.

Comparison of *CACNA1H* gene expression levels in PCC and aPGL revealed lower levels in PCC as compared to aPGL in the TCGA cohort (*P* = 0.012, Mann–Whitney *U*-test), and a trend toward lower *CACNA1H* expression in PCC vs aPGL in the Karolinska cohort (Fig. 4A). Furthermore, in the Karolinska cohort, cases with normal norepinephrine levels had lower *CACNA1H* gene expression than cases with elevated norepinephrine levels (*P* = 0.009, Mann–Whitney *U*-test; Fig. 4B). No other correlations or associations were found in the Karolinska cohort between *CACNA1H* expression and the clinical parameters.

In the Karolinska cohort, cluster 2 tumors were found to have lower *CACNA1H* expression than cluster 1 tumors (*P* = 0.001, Mann–Whitney *U*-test, Fig. 4C). Similarly, in the TCGA cohort, cluster 2 tumors had lower *CACNA1H* expression than cluster 1 tumors (*P* < 0.001, Mann–Whitney *U*-test). Given that HIF is known to bind to a *CACNA1H* promoter region (Sellak *et al.* 2014), a possible correlation between *CACNA1H* and *HIF1A* expression was investigated using TCGA data, revealing a weak correlation (*P* = 0.008, *R* = 0.199, Spearman’s rank order correlation; Fig. 4D). Furthermore, a similar analysis of *HIF2A* (*EPAS1*, related to cluster 1) revealed a correlation between *CACNA1H* and *HIF2A* (*EPAS1*) mRNA expression levels (*P* < 0.001, *R* = 0.537, *n* = 177) (Fig. 4E).

### Comparison of PPGL with *CACNA1H* variant or copy number alteration

To investigate possible functional effects of *CACNA1H* variants, we first used the TCGA dataset to compare RNA-Seq profiles between tumor groups. Altogether, 136 genes out of all 17,338 genes were significantly over-expressed, and 146 were under-expressed in the three cases with *CACNA1H* variants compared to the 174 *CACNA1H* wild-type cases. Enrichment analyses showed affected pathways related to neuronal excitability (Supplementary Fig. 3).

Additionally, copy number loss of *CACNA1H* was observed in 12 cases, while 141 were diploid and seven showed a gain. No significant difference in *CACNA1H* mRNA levels was observed between groups. In two tumors with *CACNA1H* variants, the *CACNA1H* locus was diploid and did not have any copy number loss, while the third sample´s copy number status is not known.

### Differentially expressed proteins

**Figure 5:**
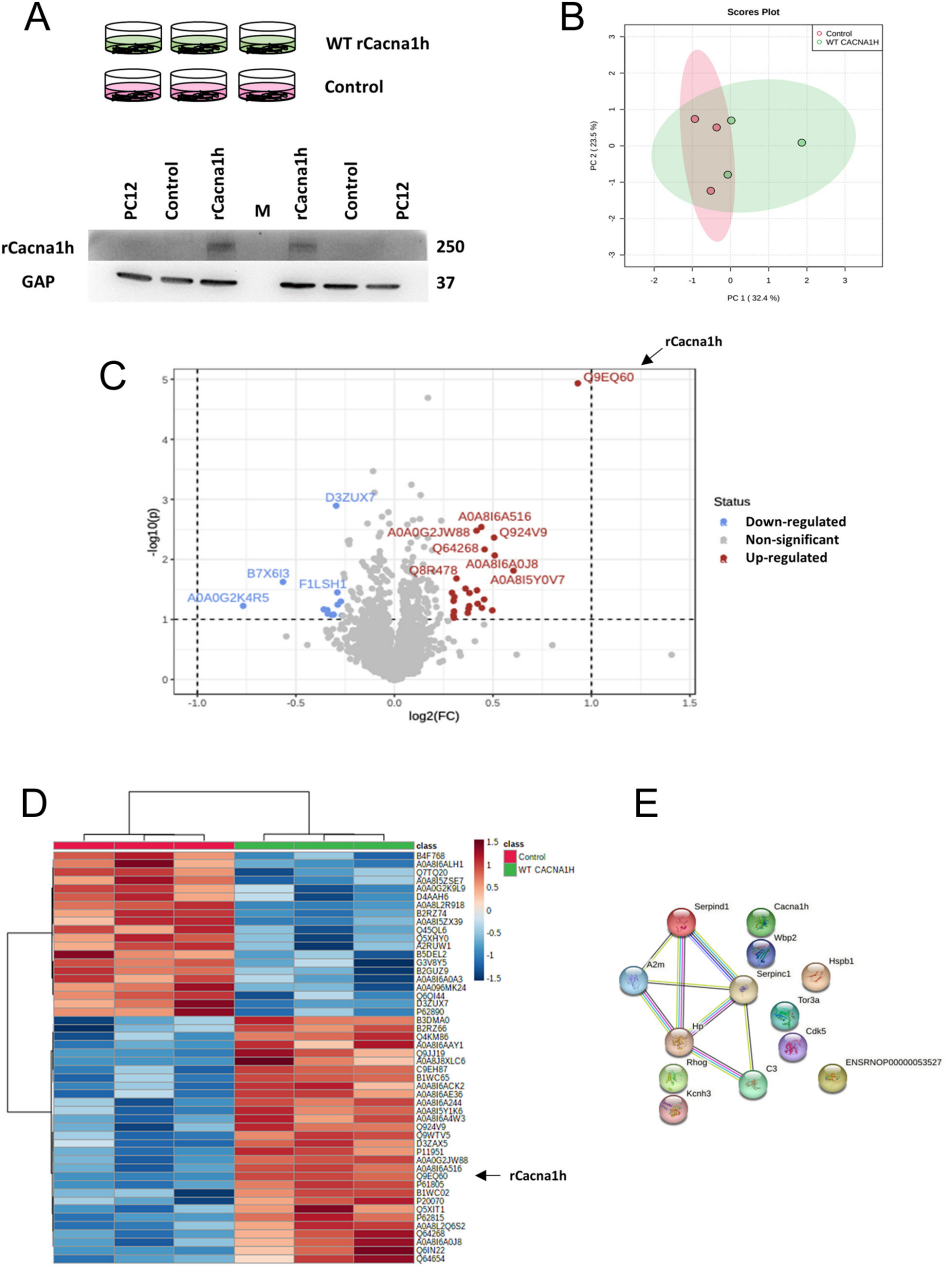
Identification of differentially expressed proteins in rat Cacna1h transfected PC12 cells. (A) PC12 rat pheochromocytoma cells were transfected in triplicate with plasmids carrying wild-type rCacna1h (WT rCacna1h) (pRP[Exp]-CAG>rCacna1h[NM_153814.2]) and vector control (control) (pRP[Exp]-CAG>Stuffer_300bp), respectively. The immunoblot below shows protein expression analysis of rCacna1h in transfected, control, and untransfected PC12 cells. GAPDH was used as a loading control. (B) Scores plot from principal component analysis (PCA) of proteomic data obtained for the six samples shown in A. (C) Proteins selected by Volcano plot of differentially expressed proteins between rCacna1h transfected and control cells. A fold-change threshold of 1.2 (*x*) and *t*-test threshold of 0.1 *(y*) were applied. Red circles represent proteins above the threshold. Fold changes and *P*-values were log-transformed. (D) Clustering of up- and down-regulated proteins illustrated as a heatmap. (E) Identification of pathways enriched for up-regulated proteins using STRING analysis.

Subsequently, we ectopically expressed wild-type rat rCacna1h and vector control in PC12 rat pheochromocytoma cells and compared the effects on global protein expression patterns and calcium currents by electrophysiology. Immunoblotting showed a very low/undetectable intrinsic level of rCacna1h in PC12 cells (Fig. 5A). Recombinant Cacna1h was transfected into PC12 cells, resulting in increased CACNA1H protein compared to the transfection control (Fig. 5A).

Using LC-MS/MS, a total of 3553 proteins were identified and quantified (Supplementary Table 7). Principal component analysis revealed the separation between samples in the two groups (Fig. 5B). Differentially expressed proteins (DEPs) were identified by statistical comparisons between rCacna1h-transfected and vector-control cells. Altogether, 335 DEPs with a *P*-value of 0.1 or below were found by *t*-test (186 up-regulated and 149 down-regulated, Supplementary Table 8). Fold-change analysis identified 59 DEPs (37 up-regulated and 22 down-regulated proteins) with a fold-change of 1.2 or more (Supplementary Table 9). Volcano plot analysis identified 37 DEPs as the most regulated proteins (26 up-regulated and 11 down-regulated, Supplementary Table 10). The clustering of DEPs is illustrated in a heatmap for the most regulated proteins (30 up-regulated and 20 down-regulated) in Fig. 5D (Supplementary Table 11). As illustrated in Fig. 5C and D, one of the top most up-regulated proteins was rCacna1h (accession number Q9EQ60), thus supporting the transfection efficiency. Among other DEPs, several have known roles in cancer (e.g. Dad1 and Brd9), adrenocortical tumors (Gnas), and cytochrome c oxidase subunits (e.g. Cox6c2 and Cox7a2l).

DEPs were assayed using the Reactome Pathway Database and String analysis. While no statistically significant associations between DEPs and annotated pathways were found when adjusting for the false discovery rate using Reactome, String analysis of up-regulated DEPs revealed enrichment of pathways related to rCacna1h and the potassium channel Kcnh3 (Fig. 5E).

### Electrophysiology

**Figure 6:**
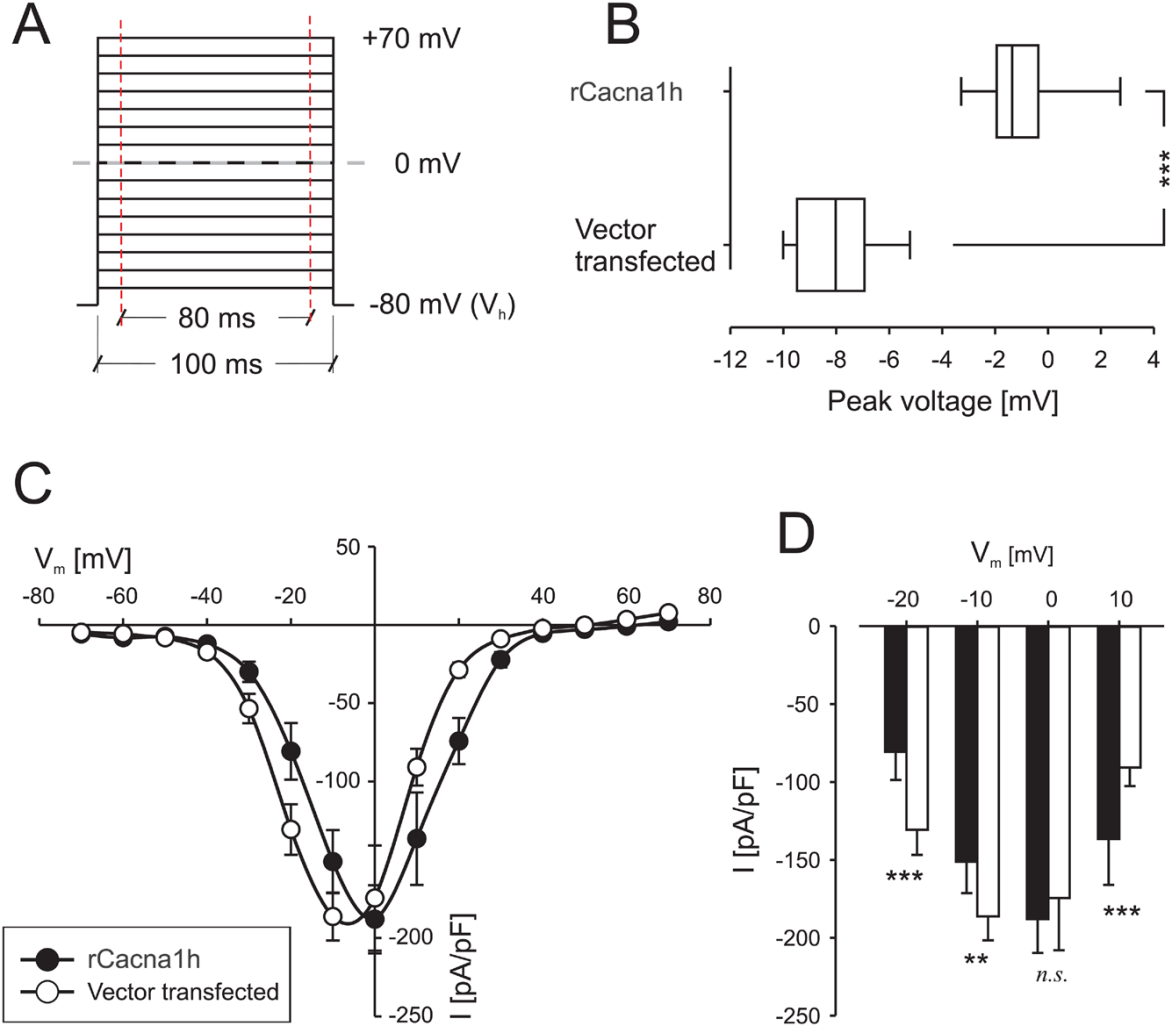
Patch-clamp experiments on rCacna1h-transfected PC12 cells. (A) Cells were voltage-clamped at −80 mV and subsequently depolarized in 10 mV incremental steps to +80 mV. Each depolarization step lasted 100 ms, and the mean current was measured omitting the first and last 10 ms of the depolarization indicated by the red lines. (B) The peak voltage was calculated by fitting each current–voltage (*I*–*V*) graph (summary presented in C) to a multi-variable regression model. (C) *I*–*V* curves for PC12 cells, controls (open circles), and rCacna1h-transfected (closed circles). (D) A summary of inward currents at −20, −10, 0, and 10 mV. Vector-transfected control PC12 cells (open bars) and rCacna1h-transfected (closed bars). Cells were randomly selected in the petri dishes in each group. **P < 0.01, ***P < 0.001, n.s., not significant.

Possible effects of *CACNA1H* overexpression were assessed by electrophysiological measurements of VOCC activity in PC12 cells using the patch-clamp technique (Fig. 6). *rCacna1h*-transfected PC12 cells and vector-transfected control cells were cultured in parallel in petri dishes 2–4 days after transfection. Cells were randomly selected in both groups’ petri dishes and clamped according to the voltage protocol presented in Fig. 6A. In total, 13 cells were analyzed in both groups, and all cells exhibited voltage-activated Ca^2+^ currents (Fig. 6C). Whole-cell currents were normalized to whole-cell capacitance (6.1 ± 0.7 pF in rCacna1h-transfected, 6.0 ± 0.5 pF in control cells, n.s.) with no detectable difference in peak current (188 ± 22 pA/pF vs 186 ± 5 pA/pF) (summary in Fig. 6D). However, *rCacna1h*-transfected PC12 cells displayed a slightly right-shifted *I*–*V* curve, with a peak current at a more positive potential. To further analyze the right shift, each *I*–*V* curve was fitted to a multi-variable regression model, and plotting the variable corresponding to the peak voltage for each group is shown in Fig. 6B.

## Discussion

In this study, the genetic background of PPGL was further explored with the identification of rare *CACNA1H* gene variants in PCC patients, including two truncating variants. Moreover, supporting the role of *CACNA1H* in PPGL development, *CACNA1H* expression was shown to be suppressed in PPGL compared with normal adrenal medulla.

For the six PCC with potentially damaging *CACNA1H* variants, mutations in other known PPGL susceptibility genes were not identified, with the exception of one case (no. 6) with a constitutional variant G2735S of unknown significance in *KMT2D* (Table 3). In WES analyses that have been performed on tumor DNA and normal DNA from cases 6 and 19 (Juhlin *et al.* 2015) and the three TCGA cases (cBioPortal) (Fishbein *et al.* 2017), additional somatic or constitutional mutations in known PPGL genes were not observed. However, we cannot exclude additional somatic PPGL gene variants in case E2, and additional genes may be added to the growing list of PPGL genes.

Six potentially damaging *CACNA1H* variants were found by combined analyses of the Karolinska cohort and the TCGA database. In the TCGA cohort, the observed frequency of *CACNA1H* variants was 1.7% in PPGL and 2% in PCC. Taken together, the TCGA and Karolinska cohorts suggest a frequency of *CACNA1H* variants of 1.8% in PPGL and 2.2% in PCC.

In addition, Flynn *et al.* reported a somatic missense variant R2322Q/c.6965G>A predicted as benign in a PCC case with an *NF1* mutation (Flynn *et al.* 2015).

Several observations support the role of *CACNA1H* variants in PPGL. Four of the detected *CACNA1H* variants were not present in gnomAD, and two were present at very low frequencies. Three of the four missense variants were predicted as damaging by multiple programs (Adzhubei *et al.* 2010), and the two frameshift deletions are also expected to be damaging. The missense variant L1447M demonstrated damaging predictions from two computational prediction tools, contrasting with benign predictions from alternative computational analyses. The variant G1064R is annotated in ClinVar with uncertain pathogenicity. Hence, elucidation of their pathogenicity awaits scrutiny through functional assays. Furthermore, all missense variants affect amino acids that are evolutionarily preserved in orthologs (Supplementary Fig. 1). Two of the missense variants have been reported in conditions affecting the adrenal cortex. Constitutional G1064R has been reported in a family with primary hyperaldosteronism, and the somatically occurring variant I1430T was observed in three aldosterone-producing adrenocortical adenomas. Both variants have been investigated *in vitro,* showing functional consequences of the investigated variant with effects on aldosterone production and gene expression (Roomp 2016, Nanba *et al.* 2020). Additional *CACNA1H* variants have been reported in primary hyperaldosteronism and aldosterone-producing adrenocortical adenoma (Fig. 1) (Wu *et al.* 2021, Tseng *et al.* 2022, Liu *et al.* 2021, Daniil *et al.* 2016). Of these, M1549V and M1549I are located close to W1530S observed in the present study, which both affect the pore-forming segment 6 in repeat III (Fig. 1). Similarly, L1447M and I1430T affect segment 5 of repeat III, which is also involved in the pore for calcium-flux. Also, V1937M and M1549V have been shown to have functional consequences *in vitro* and in mouse models, respectively (Gürtler *et al.* 2020, Tseng *et al.* 2022, Seidel *et al.* 2021). Hence, there is reason to predict the functional effects of the *CACNA1H* variants observed in PCC, especially for those assessed in primary hyperaldosteronism and aldosterone-producing adenoma, respectively. At the same time, none of the three cases with demonstrated constitutional *CACNA1H* variants presented signs of primary aldosteronism. This could possibly be related to reduced penetrance or the influence of other factors influencing the development of PCC or primary aldosteronism in carriers of *CACNA1H* variants. Additional investigations into the functional consequences of the detected variants could possibly clarify the role of *CACNA1H* in PPGL development.

*CACNA1H* gene expression was demonstrated by multiple methodologies to be generally suppressed in PPGL tumor tissue in comparison to the non-tumorous adrenal tissue consisting of both the adrenal medulla and cortex. Immunohistochemistry allows precise tissue observations, and in our cohort, the tumor tissue was generally less intense than the normal medulla, demonstrating that the protein is downregulated in the tumor.

In PC12 rat cells, CACNA1H is upregulated following cellular stress such as chronic hypoxia (Mahapatra *et al.* 2012), and hypoxia has been shown to increase the expression of Ca_V_3.2 and its function due to the binding of HIF1A to the *CACNA1H* promoter region (Sellak *et al.* 2014). In our study, *HIF1A* and *CACNA1H* expressions were weakly correlated, and cluster 1 tumors, characterized by pseudohypoxia, were found to have higher expression of *CACNA1H*. In agreement, the expression of *HIF2A* (*EPAS1*) on which the pseudohypoxia phenotype is based, was correlated with *CACNA1H* mRNA levels. Based on mRNA expression profiles, five of the *CACNA1H* variant cases were cluster 2, while one was cluster 1, although without typical aggressive features or high methylation phenotype.

Additional mechanisms of *CACNA1H* suppression may therefore be operative in PPGL. The observation of correlations between DNA methylation density across the *CACNA1H* gene and its mRNA expression could suggest epigenetic suppression as an additional mechanism involved in the tumor-specific suppression. Of interest, *CACNA1H* has been reported as a differentially methylated gene distinguishing adrenocortical tumor subgroups (Clay *et al.* 2019).

The hypermethylator phenotype in PPGL recognizes the M1 subgroup with hypermethylation of multiple promoter CpGs as compared to non-M1 tumors, including *CACNA1H* (Letouzé *et al.* 2013). The methylation of three sites in the promoter region reported in Letouze *et al.* was not associated with decreased *CACNA1H* expression among M1 tumors (Letouzé *et al.* 2013). While the corresponding sites 45−47 are located in a region of generally low methylation when looking at all 177 PPGL, increased methylation for multiple sites of the gene is associated with increased *CACNA1H* expression (Fig. 3). In addition, *CACNA1H* levels were higher in PPGL with an M1 hypermethylator phenotype.

Gene enrichment analyses comparing tumors with *CACNA1H* variants and wild-type *CACNA1H* revealed a set of affected pathways (Supplementary Fig. 3). Interestingly, since CACNA1H is a regulator of neuronal excitability, this could potentially indicate a functional difference between the two groups.

In functional studies in HEK293 cells of the recurrent *CACNA1H* variant M1549V, reported in primary hyperaldosteronism, loss of normal channel inactivation and activation at different potentials were observed (Scholl *et al.* 2015). Depolarization through increased intracellular calcium ion concentrations in PC12 cells has been linked to changes in overall morphology, suggesting that an altered influx of calcium in PPGLs could influence tumoral behavior (Starikova *et al.* 2000). We, therefore, performed functional experiments overexpressing rat CACNA1H (rCacna1h) specifically to address if this particular calcium channel influences the electrophysiologic state of PC12 rat pheochromocytoma cells, as well as the overall proteome of these cells. Some of the observed DEPs are of particular interest for the adrenal gland. Among these can be mentioned *GNAS Complex locus* (*GNAS*) which is known to be mutated in a subset of adrenocortical tumors. Furthermore, we observed upregulation of cytochrome c oxidase subunits (e.g. Cox6c2 and Cox7a2l), which are part of the mitochondrial electron transport chain of central importance in PPGL. The patch-clamp experiments showed that the membrane potential for the peak current of Ca2+ currents was right shifted in PC12 cells overexpressing rCacna1h compared to control cells. However, no difference was seen in peak current or integrated current. Possible explanations for these unexpected findings are that transfection with rCacna1h affects the expression of other regulatory factors or Ca2+ channels. Another possible explanation is that in a population with a less than 100% transfection ratio, some of the randomly patched cells may be non-transfected, thereby underestimating the effect of the rCacna1h-transfected group. Indeed, our findings suggest that rCacna1h over-expressing PC12 cells exhibited a shift in the membrane potential for Ca2+ currents as well as changes in the proteome.

T-type low VOCCs lower the threshold of the action potential, thereby facilitating the cells to respond to sympathetic stimulation at lower voltage levels but also creating a continuous release of catecholamines at resting potential (Marcantoni *et al.* 2008). Scholl *et al.* compared *CACNA1H* missense mutations observed in primary aldosteronism to wild-type *CACNA1H* in a patch clamp experiment and observed that the mutated cells left-shifted, thus obtaining a lower threshold for activation and an almost ten-fold slower inactivation than wild-type channels, thereby increasing the secretion of aldosterone (Scholl *et al.* 2015). Conversely, when we overexpressed *CACNA1H* in PC12 cells, we observed a rightward shift in our patch clamp experiment. This shift suggests that the cells were less likely to respond to normal triggers because it takes a stronger electrical signal to reach the threshold required for activation. Hence, the reduced *CACNA1H* levels in tumors compared to normal and the truncating variants in our study could potentially confer a similar response to membrane potential as observed in primary aldosteronism, although it is important to emphasize that comparing site-directed mutagenesis studies and enforced overexpression experiments can be challenging.

The expression of CACNA1H in the adrenal coincides with tyrosine hydroxylase, a marker indicating chromaffin cells with catecholamine secretion (Levitsky & López-Barneo 2009). However, in normal adrenal medullary tissue, several types of calcium channels (L, N, P/Q, R, and T) are involved in chromaffin cell excitability and exocytosis of catecholamines (García-Palomero *et al.* 2000). In our study, we observed associations between *CACNA1H* expression and norepinephrine, as well as cluster 1 vs 2 phenotypes, which could theoretically reflect the difference in norepinephrine levels between cluster 1 and 2 cases and/or a causative relation. Cluster 1 PPGL exhibits an immature secretion apparatus with higher secretion of catecholamines (Eisenhofer *et al.* 2011). At the same time, we showed that *in vitro* expression of *CACNA1H* led to a higher threshold for activation. This discrepancy could partly be explained by the diversity in channels involved in the secretion (such as L, N, P/Q, R, and T). To fully explain the relationships between the different calcium channels in chromaffin cells, specifically CACNA1H, further analyses at the individual cell level would have to be made.

In conclusion, the findings suggest a role for *CACNA1H/*Ca_V_3.2 in the development of PPGL. Genetic *CACNA1H* variants are reported, and *CACNA1H*/Ca_V_3.2 expression was shown to be generally suppressed in pheochromocytoma/abdominal paragangliomas compared to non-tumorous adrenal medulla, with possible relation to epigenetic inactivation and influence of hypoxia. The observations propose a possible overlap in the etiology of adrenocortical tumors and provide the rationale for further studies into the role of *CACNA1H* in the development of PPGL.

## Supplementary Materials

Supplementary Materials

Supplementary Figure S1

Supplementary Figure S2. CACNA1H immunohistochemical expression in two PPGLs with CACNA1H variants.

Supplementary Figure S3. Enrichment analyses comparing CACNA1H variants to CACNA1H wild-type cases. Data from cbioportal/TCGA and analyses/graphs were made using WebGestalt.

Supplementary Tables

## Declaration of interest

The authors declare that there is no conflict of interest that could be perceived as prejudicing the impartiality of the research reported.

## Funding

The study was financially supported by the Swedish Research Council, the Swedish Cancer Society, the Gustav V Jubilee Foundation, Stockholm county council (ALF), and Karolinska Institutethttp://dx.doi.org/10.13039/501100004047.

## Ethics approval and consent to participate

All tissue samples were collected with informed consent, and the study of the tissue material was approved by the Swedish Ethical Review Authority.

## Data availability

The mass spectrometry proteomics data have been deposited to the ProteomeXchange Consortium (http://proteomecentral.proteomexchange.org) via the PRIDE partner repository (Perez-Riverol *et al.* 2022) with the dataset identifier PXD040446.
