# Supplementary material for: Genetic variants and down-regulation of CACNA1H in pheochromocytoma
Source: Endocr Relat Cancer. 2024 Jul 8;31(9):e230061. doi: 10.1530/ERC-23-0061 (PMC11301417; doi:10.1530/ERC-23-0061)
Supplement: Supplementary Materials [file supplementary_material.pdf]

Supplementary Document

Materials and Methods

Clinical assessment of catecholamine levels in the Karolinska cohort

The Karolinska cohort comprises patients where urine samples were used for the measurement and others where metanephrines were measured in plasma, contingent upon their year of diagnosis. We therefore analyzed catecholamine categories as elevated or normal. By employing such a categorization, we aimed to streamline the analysis and enhance our ability to discern clinically relevant thresholds and patterns, leveraging the larger sample size available.

For measurements of catecholamines in serum blood was drawn from fasting patients using a standardized procedure and collected in cooled sodium-heparin tubes and sent on ice blocks for immediate centrifugation. For measurements in urine, patients contributed 24-hour urinary samples. Measurements were conducted as part of clinical routine work-up using standardized methodology and at the Department of Clinical Chemistry, Karolinska University Hospital, Stockholm, Sweden.

Sanger sequencing

Ninety-five PPGL were screened for variants in “hotspot” areas of exons 16, 17, 22, 25 and 34 denoting regions in which we observed *CACNA1H* variants either from the former WES study of the discovery cohort (Juhlin *et al.* 2015), the TCGA database and/or findings from screening at the Clinical Genetics Department at the Karolinska University Hospital. The analyses used five different primer pairs detailed in Supplementary Table S4. Primers for exons 16 and 22 were commercially available: Hs00581474_CE (exon 16, Thermo Fisher) and Hs00553816_CE (exon 22, Thermo Fisher). The remaining three primer pairs were designed in-house. DNA in concentration 25 ng/µl was used. PCR was run with Platinum™ II Hot-Start PCR Master Mix and primers using touch down 50-60 ºC. ExoSAP-IT™ PCR Product Cleanup Reagent (Thermo Fisher) was used to eliminate background signalling. Sanger sequencing was performed by the KIgene core facility at Karolinska Institutet. Sequences were analysed using Chromas 2.6.6 1998-2018 and Genome Compiler Corporation, Los Altos, CA, 2015. Variants were then assessed using Ensembl Genome Browser, MutationTaster and Polyphen2. All variants were denoted according to HGVS nomenclature and using the reference sequence NM_021098.3.

Quantitative real-time polymerase chain reaction (qRT-PCR)

CACNA1H mRNA expression was successfully analysed by qRT-PCR in 83 PPGL and 8 normal adrenal tissue samples. Experimental procedures and data analyses were performed according to TaqMan 2X Universal PCR Master Mix user guide for gene expression quantification. Total RNA was converted to cDNA using High-Capacity RNA-to-cDNA kit (Thermo Fisher). cDNA diluted at 25 ng/µl was used to determine relative mRNA expression for *CACNA1H* (Assay ID: Hs00234934, Thermo Fisher) normalized to *B2M* (Assay ID: Hs99999907, Thermo Fisher) used as a housekeeping gene. Samples were run in triplicates using an ABI 7900HT Real-time PCR System (Applied Biosystems). Data was analysed in Excel (Microsoft Office). A mean Ct value was calculated for each sample and final results were obtained after calculating 2^-deltaCt.

Immunohistochemistry

CACNA1H protein expression was investigated by immunohistochemistry in 29 PPGL cases (20 PCC and 9 PGL). As controls, tissue samples of normal testis, normal pancreas, a Pan-NET and normal adrenal were analyzed in parallel. Twelve of the PPGL samples had normal adrenal medulla and 18 samples had normal adrenal cortex present in the same slides which were used for comparison.

Slides were deparaffinized using xylene and rehydrated in ethanol. Citrate buffer (pH 6) was used for antigen retrieval and peroxidase blocking was performed using hydrogen peroxidase. Following BSA blocking slides were incubated with primary antibody against the T-type Ca^{++} CP α1H (G-10, sc-377510, Santa Cruz Biotechnology, Inc.) at dilution 1:400. The antibody concentration was selected based on staining of control slides with normal pancreas and testis. Slides were then incubated with the secondary antibody ImmPRESS peroxidase reagent anti- mouse IgG (# MP-7402, VECTOR Laboratories) followed by DAB chromogen ImmPACT DAB (SK-4105, VECTOR Laboratories).

Slides were counterstained with hematoxylin and subsequently evaluated by a pathologist (CCJ) blinded to clinical and genetic data. Cytoplasmatic immunoreactivity was graded as: Minimal or no staining (0); Mixed staining pattern with negatively and positively stained cells (+/-); Weak staining intensity (+); or Strong staining intensity (++).

***In silico* analysis of genetic variants and Enrichment analyses**

Detected variants were analyzed using Alamut Visual Plus v.1.7 (SOPHiA GENETICS) and analyses from the following analyses tools are included: Mutation Taster (v2021) with tree

vote (deleterious/benign); Polymorphism Phenotyping v2 (PolyPhen2) score; Combined Annotation Depletion v 1.6 (CADD) phred score; Sorting Intolerent from Tolerent v6.2.0 (SIFT) score; Align Grantham Variation Grantham Difference v2007 (align-GVGD) class; and Grantham Distance. Allele frequencies and possibly variant IDs were obtained for gnomAD using Alamut Visual Plus v.1.7. In addition, Mutation Taster was used separately for predictions of the splice variants.

Differentially expressed genes between *CACNA1H* variant and *CACNA1H* wild-type cases were identified in cbioportal (https://www.cbioportal.org/). Enrichment analyses including Gene Set Enrichment analyses (GSEA) and Over-representation analyses (ORA) were performed using WebGestalt (https://www.webgestalt.org) (liao *et al*. 2019). In the GSEA 17,338 genes and their Log2ratio were analyzed and in the ORA all significant DEGs (n = 278) were included.

Cell culture, plasmids and transfection

The established rat adrenal pheochromocytoma cell line PC12 was purchased from Sigma-Aldrich (#88022401, ECACC). Cells were cultured in RPMI 1640 Medium (Gibco, #A1049101) supplemented with 10% Horse Serum (#26050088, Thermo Fisher) and 5% fetal Bovine Serum (#26140079, Gibco) in type IV collagen coated flasks. Custom made plasmids were purchased from VectorBuilder GmbH, Germany. The plasmid VB191122-3128xxz including the rat rCacna1h gene in the expression vector pRP[Exp]-CAG>Stuffer_300 bp was used for over-expression of rCacna1h. The plasmid VB190607-1105xft constituting of the same expression vector without rCacna1h was used as control. For transfection, 300,000 cells were plated in each well and cultured for 24 h followed by transfection with 2 ug of plasmid per well using lipofectamine LTX (#A12621, Invitrogen) and harvesting after 48 h.

Expression of rCacna1h was analysed in protein extracts from cell lysates of transfected and untransfected PC12 cells using immunoblotting according to previously described methodology (Liu *et al*. 2022). Antibodies targeting rCacna1h (#ACC-025, Alomone labs, rabbit, 1:200) and GAPDH (#5174, CST, rabbit, 1:2,000) were used as primary antibodies, and anti-rabbit (#7074, CST, 1:1000) were employed as secondary antibodies.

Mass spectrometry and data analyses

Protein was harvested from PC12 cells transfected with wild-type rCacna1h plasmid and control vector plasmid, respectively, and liquid chromatography with tandem mass spectrometry (LC-MS/MS) was performed at the Proteomics Biomedicum core facility,

Karolinska Institutet. Totally six samples were analyzed including three rCacna1h transfected and three vector control. The experimental procedures and data analysis largely followed previously reported procedures (Shafi *et al*. 2023) with modifications, employing the typical isobaric labeling based quantification approach.

Protein extracts from PC12 cells transfected with wild-type rCacna1h plasmid and control vector plasmid, respectively, were profiled by LC-MS/MS using the isobaric labeling based quantification approach. Cell pellets were solubilized with 80 µL of 4 M urea and 1% ProteaseMAX (Promega) in 50 mM ammonium bicarbonate (AmBic), pH 8.5 with 1 µL of 100x protease inhibitor cocktail (Roche) following sonication in water bath for 5 min and probe sonicated with VibraCell probe (Sonics & Materials, Inc.) for 40 s, with pulse 2/2, at 20% amplitude. Protein concentration was measured by BCA assay (Thermo Scientific). An aliquot of 25 µg samples was transferred to a new tube and equalized with 50 mM AmBic to a total volume of 70 µL. Proteins were reduced with addition of 5 µL of 100 mM dithiothreitol (Sigma) at 37°C for 60 min and alkylated with 15 µL of 100 mM iodoacetamide for 30 min at room temperature (RT) in the dark. Sequencing grade trypsin (Promega) was added in an enzyme to protein ratio of 1:50 (5 µL of 0.1 µg/µL) and digestion was carried out over night at 37°C. The digestion was stopped with 5 µL cc. formic acid, incubating the solutions at RT for 5 min. The sample was cleaned on a C18 Hypersep plate with 40 µL bed volume (Thermo Fisher Scientific), and dried in a vacuum concentrator (Eppendorf). Biological samples were labeled with TMT-6plex reagents in random order adding 100 µg TMT-reagent in 30 µL dry acetonitrile (ACN) to each digested sample resolubilized in 70 µL of 50 mM triethylammonium bicarbonate (TEAB) and incubating at RT for 2 h. The labeling reaction was stopped by adding 11 µL of 5% hydroxylamine and incubated at RT for 15 min before combining them in one vial.

TMT-labeled sample was reconstituted in solvent A and approximately 2 µg were loaded on a 50 cm long EASY-Spray C18 column (Thermo Fisher Scientific) connected to an Ultimate 3000 nanoUPLC system (Thermo Fisher Scientific). The peptides were eluted using a 120 min long gradient: 4-26% of solvent B (98% acetonitrile, 0.1% FA) in 120 min, 26-95% in 5 min, and 95% of solvent B for 5 min at a flow rate of 300 nL/min. Mass spectra were acquired on a Q Exactive HF hybrid quadrupole Orbitrap mass spectrometer (Thermo Fisher Scientific) ranging from *m/z* 375 to 1700 at a resolution of R=120,000 (at *m/z* 200) targeting $5x10^6$ ions for maximum injection time of 80 ms, followed by data-dependent higher-energy collisional dissociation (HCD) fragmentations of precursor ions with a charge state 2+ to 8+, using 45 s dynamic exclusion. The tandem mass spectra of the top 18 precursor ions were acquired with

a resolution of R=60,000, targeting $2x10^5$ ions for maximum injection time of 54 ms, setting quadrupole isolation width to 1.4 Th and normalized collision energy to 33%.

Acquired raw data files were analyzed using Proteome Discoverer v2.5 (Thermo Fisher Scientific) with MS Amanda 2.0 (doi.org/10.1021/pr500202e) search engine against rat protein database (UniProt 10116, 54,422 entries). A maximum of two missed cleavage sites were allowed for full tryptic digestion, while setting the precursor and the fragment ion mass tolerance to 10 ppm and 0.02 Da, respectively. Carbamidomethylation of cysteine was specified as a fixed modification, while TMT6plex on lysine and N-termini, oxidation on methionine as well as deamidation of asparagine and glutamine were set as dynamic modifications. Initial search results on proteins were filtered with 5% FDR using Percolator node in Proteome Discoverer. Quantification was based on the TMT-reporter ion abundances.

After normalization, differentially expressed proteins between rCacna1h transfected and control samples were identified. Univariate analysis of two-group data included two-tailed t-tests (threshold for p-value 0.1), fold change analysis (threshold for fold change 1.2), and volcano plot (threshold for fold change 1.2 and t-test p-value 0.1). Variance in the data between samples was illustrated by unsupervised principal component analysis (PCA). Hierarchical clustering was done and differentially expressed proteins highlighted in a heatmap.

Protein ontology analyses

In order to assess any type of overrepresentation of specific pathways among proteins that were found to be differentially expressed between rCacna1h-transfected and vector control PC12 cells, the Reactome Pathway Database was used (https://reactome.org/). The top 150 proteins with significant p-values suggestive of a statistically significant difference between groups that were assayed, as well as a separate analysis with the 59 Differentially expressed proteins (DEP) with a fold change of 1.2 or more between groups. For all analyses, *R. norvegicus* was selected as the species model. Moreover, String analysis was applied to identify enriched pathways among up-regulated and down-regulated DEPs.

Electrophysiology

For electrophysiological experiments, the analyses largely followed previously published experimental procedures (Lu *et al.* 2010) with modifications. Cells were seeded into Petri dishes (Nunc, Roskilde, Denmark) and incubated at 37 °C and 5% CO_2 overnight. Voltage-

operated calcium channels (VOCC) activity was recorded using the patch-clamp technique (Hamill *et al*. 1981) with a HEKA EPC-10 patch-clamp amplifier (HEKA Elektronik, Ludwigshafen, Germany). The extracellular solution (i.e., bath solution) contained the following: 138 mM NaCl, 5.6 mM KCl, 20 mM $BaCl_2$, 1.2 mM $MgCl_2$, and 5 mM HEPES at a pH of 7.4. For whole-cell recordings of Ca^{2+} currents, the intracellular-like solution (i.e., pipette solution) consisted of the following: 135 mM CsCl, 1 mM $MgCl_2$, 2 mM $CaCl_2$, 10 mM EGTA, 1 mM MgATP, and 5 mM HEPES (pH 7.15 with CsOH). In all whole-cell recordings, R_S <40 MΩ and cell capacitance were updated between every voltage-protocol cycle. Cells were voltage-clamped at -80 mV and subsequently depolarized for 100 ms in +10 mV steps until +80 mV. Capacitive and leakage current subtraction using the P/N method and channel current traces were displayed according to the convention that downward deflection denotes inward currents. Pipettes were pulled from borosilicate using a P-2000 laser pipette puller (Sutter Instrument, Novato, CA, USA), and they had a resistance between 2 and 4 MΩ. All the experiments were performed at a room temperature of +22 °C.

Statistical analyses and illustrations

Results from mutational and expressional studies were used for statistical analysis together with previously published but updated clinical characteristics and expressional profiles from (Stenman *et al.* 2019). IBM SPSS Statistics Version 25 and 26 were used for statistical analyses and graphic work. Correlations were assessed by Spearman's Rank Order Correlation and for comparisons between groups the Mann-Whitney U-test or Pearson´s chi-square tests were performed. Illustrations were made using Inkscape 0.92 and PowerPoint (Microsoft Office). Outliers were omitted manually for enlarged illustrations of correlations. P-values < 0.05 were considered as statistically significant, with the exception of correlations to CG methylation where $P < 0.01$ was applied because of multiple testing. R-values < 0.4 were considered weak or of no significance.

Patient presentations of the three cases with constitutional *CACNA1H* variants

Case no 6 is a female with no reported family history of PPGL, who was diagnosed and operated for a pheochromocytoma at age 44. At the time, the patient presented with attacks of headaches, visual disturbance and palpitations. She also had intractable hypertension for 10-15 years back, with the highest measure 205/125. Biochemical investigations showed raised norepinephrine in urine. The histopathological examination showed a 2 cm PCC with no evidence of malignant behaviour. The patient was diagnosed with breast cancer over 20 years after the PCC operation. She was also treated for multinodular toxic goitre and developed hypothyroidism after initial treatment. After 31 years of follow-up, the patient is still alive with no evidence of disease, and no signs of hyperaldosteronism was seen. Through whole exome sequencing carried out in a research setting, a constitutional *KMT2D* mutation (G2735S) was revealed (Juhlin *et al*. 2015).

Case no 19 is a male, who was diagnosed and operated at the age of 37. Symptoms included vomiting, vertigo and considerable raises in blood pressure. No family history of PPGL was acknowledged. The histopathological examination showed a 10 cm PCC with a PASS score of 1 and no evidence of malignant disease. Prior to the PCC, the patient had also been operated for seminoma. He was also diagnosed with neurofibromas but tested negative at *NF1* mutation screening. In recent years, the patient was diagnosed with gall stones and has also been bothered by palpitations and was diagnosed with short ventricular tachycardia and premature ventricular contractions which was treated with calcium blockers. The patient is currently alive 27 years after the PCC operation with no evidence of recurrent disease or signs of hyperaldosteronism.

Case no E2 was a 51-year old male, with no prior health issues, who presented with chest pain and neurological symptoms during a walk. He was in cardiac chock and had pulmonary oedema. Troponin as well as metanephrines and 3-methoxythyramin were raised. CT scan revealed a 4.5 cm tumor of the adrenal medulla. Potassium levels were normal. He was put in ECMO for life support but deceased. The histopathological report from autopsy showed a PCC with Ki-67-index of 1%, necrotic tissue and signs of ruptured PCC with secondary bleedings. Histology did not reveal any signs of hyperaldosteronism. Whole-exome sequencing (WES) of a sample of normal myocardium was performed at the clinical genetics department. As of now, all PPGL patients are routinely recommended a referral for whole-genome sequencing (WGS) or WES (in the case of FFPE material) of germline DNA with

analysis of an established gene panel for endocrine tumors including presently known clinically actionable PPGL susceptibility genes (Muth *et al*. 2019) and a subset of genes previously coupled to tumors of the adrenal cortex and other endocrine tumors (including *CACNA1H*) (Supplementary Table S3). In short, WES data was generated and small nucleotide variants (SNV) were detected and evaluated as previously described (Lindstrand *et al*. 2019). In Case E2 analysis of 59 genes of the endocrine tumor panel (Supplementary Table S3) showed absence of mutations in known PPGL susceptibility genes.

References

Hamill OP, Marty A, Neher E, Sakmann B & Sigworth FJ 1981 Improved patch-clamp techniques for high-resolution current recording from cells and cell-free membrane patches. *Pflugers Archiv : European Journal of Physiology* 391 85-100.

Juhlin CC, Stenman A, Haglund F, Clark VE, Brown TC, Baranoski J, Bilguvar K, Goh G, Welander J, Svahn F, Rubinstein JC, Caramuta S, Yasuno K, Günel M, Bäckdahl M, Gimm O, Söderkvist P, Prasad ML, Korah R, Lifton RP & Carling T 2015 Whole-exome sequencing defines the mutational landscape of pheochromocytoma and identifies KMT2D as a recurrently mutated gene. *Genes Chromosomes Cancer* 54 542-554.

Liao Y, Wang J. Jaehnig E, Shi Z & Zhang B 2019 WebGestalt 2019: gene set analysis toolkit with revamped UIs and APIs. *Nucleic Acids Research* 47 W199-W205.

Lindstrand A, Eisfeldt J, Pettersson M, Carvalho CMB, Kvarnung M, Grigelioniene G, Anderlid BM, Bjerin O, Gustavsson P, Hammarsjö A, Georgii-Hemming P, Iwarsson E, Johansson-Soller M, Lagerstedt-Robinson K, Lieden A, Magnusson M, Martin M, Malmgren H, Nordenskjöld M, Norling A, Sahlin E, Stranneheim H, Tham E, Wincent J, Ygberg S, Wedell A, Wirta V, Nordgren A, Lundin J & Nilsson D 2019 From cytogenetics to cytogenomics: whole-genome sequencing as a first-line test comprehensively captures the diverse spectrum of disease-causing genetic variation underlying intellectual disability. *Genome Medicine* 11 68.

Lu M, Bränström R, Berglund E, Höög A, Björklund P, Westin G, Larsson C, Farnebo LO & Forsberg L 2010 Expression and association of TRPC subtypes with Orai1 and STIM1 in human parathyroid. *Journal of Molecular Endocrinology* 44 285-294.

Muth A, Crona J, Gimm O, Elmgren A, Filipsson K, Stenmark Askmalm M, Sandstedt J, Tengvar M & Tham E 2019 Genetic testing and surveillance guidelines in hereditary pheochromocytoma and paraganglioma. *Journal of Internal Medicine* 285 187-204.

Shafi AM, Végvári Á, Zubarev RA & Penha-Gonçalves C 2023 Brain endothelial cells exposure to malaria parasites links type I interferon signalling to antigen presentation, immunoproteasome activation, endothelium disruption and cellular metabolism. *Frontiers in Immunology* 14 1149107.

Stenman A, Svahn F, Hojjat-Farsangi M, Zedenius J, Söderkvist P, Gimm O, Larsson C & Juhlin CC 2019 Molecular profiling of pheochromocytoma and abdominal paraganglioma stratified by the PASS algorithm reveals chromogranin B as associated with histologic prediction of malignant behavior. *The American Journal of Surgical Pathology* 43 409-421.
