# Supplementary material for: Genetic variants and down-regulation of CACNA1H in pheochromocytoma
Source: Endocr Relat Cancer. 2024 Jul 8;31(9):e230061. doi: 10.1530/ERC-23-0061 (PMC11301417; doi:10.1530/ERC-23-0061)
Supplement: Supplementary Figure S1 [file supplementary_figure_1.pdf]

G1064R

▼

Amino acid	1061	1062	1063	1064	1065	1066	1067
Human	T	P	N	G	H	L	E
Rhesus	T	P	N	G	H	L	E
Mouse	T	P	N	G	H	L	E
Dog	T	P	N	G	H	L	E
Elephant	T	P	N	G	H	L	E
Chicken	T	P	N	G	H	L	E
X-tropicalis	T	P	N	G	H	L	D
Zebrafish	S	A	N	G	H	V	D
Lamprey							

Q1159P

▼

Amino acid	1156	1157	1158	1159	1160	1161	1162
Human	R	R	G	Q	C	G	E
Rhesus	R	R	S	Q	C	G	E
Mouse	R	R	S	Q	C	G	E
Dog	R	R	S	Q	C	G	E
Elephant	R	R	S	Q	C	G	E
Chicken	K	K	N	Q	S	G	E
X-tropicalis	K	K	S	Q	S	G	E
Zebrafish	R	K	D	T	S	G	E
Lamprey	P				P	G	G

I1430T

▼

Amino acid	1427	1428	1429	1430	1431	1432	1433
Human	L	R	P	I	G	N	I
Rhesus	L	R	P	I	G	N	I
Mouse	L	R	P	I	G	N	I
Dog	L	R	P	I	G	N	I
Elephant	L	R	P	I	G	N	I
Chicken	L	R	P	I	G	N	I
X-tropicalis	L	R	P	I	G	N	I
Zebrafish	L	R	P	I	G	N	I
Lamprey	L	K	P	I	G	N	I

L1447M

▼

Amino acid	1444	1445	1446	1447	1448	1449	1450
Human	F	G	I	L	G	V	Q
Rhesus	F	G	I	L	G	V	Q
Mouse	F	G	I	L	G	V	Q
Dog	F	G	I	L	G	V	Q
Elephant	F	G	I	L	G	V	Q
Chicken	F	G	I	L	G	V	Q
X-tropicalis	F	G	I	L	G	V	Q
Zebrafish	F	G	I	L	G	V	Q
Lamprey	F	G	I	L	G	V	Q

W1530S

▼

Amino acid	1527	1528	1529	1530	1531	1532	1533
Human	H	N	P	W	M	L	L
Rhesus	H	N	P	W	M	L	L
Mouse	H	N	P	W	M	L	L
Dog	H	N	P	W	M	L	L
Elephant	H	N	P	W	M	L	L
Chicken	H	N	P	W	M	L	L
X-tropicalis	H	N	P	W	M	L	L
Zebrafish	H	N	P	W	M	L	L
Lamprey	H	N	P	W	M	L	L

Supplementary Figure S1. Conservation of CACNA1H amino acids sequences in orthologs at the positions of detected missense variants (arrows) and surrounding amino acids. Data collected from UCSC genome browser.
