# Supplementary material for: Genetic variants and down-regulation of CACNA1H in pheochromocytoma
Source: Endocr Relat Cancer. 2024 Jul 8;31(9):e230061. doi: 10.1530/ERC-23-0061 (PMC11301417; doi:10.1530/ERC-23-0061)
Supplement: Supplementary Figure S2. CACNA1H immunohistochemical expression in two PPGLs with CACNA1H variants. [file supplementary_figure_2.pdf]

A

B

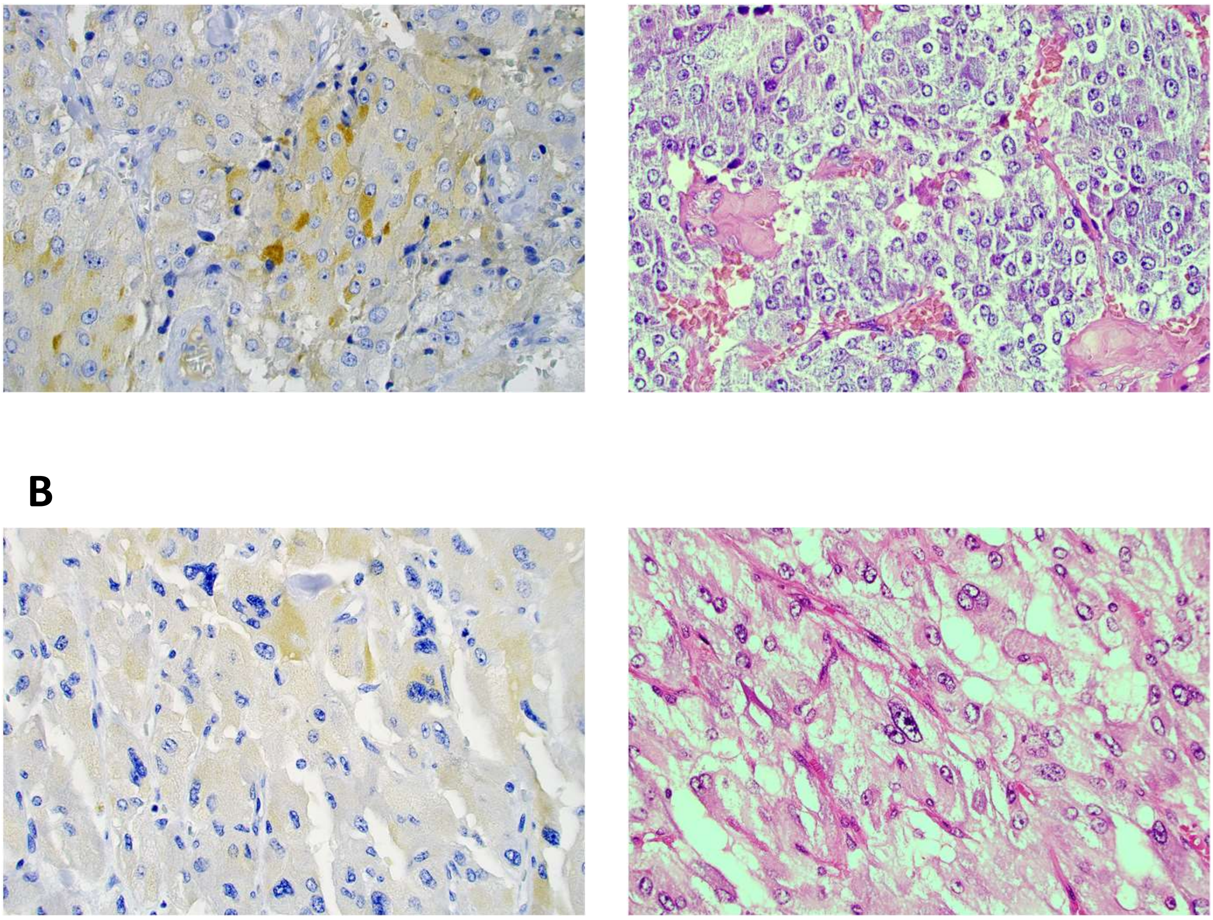

Supplementary Figure S2. **CACNA1H immunohistochemical expression in two PPGLs with *CACNA1H* variants.** (A) Immunohistochemical analysis of CACNA1H protein expression in case 6. PCC sample showed varied cytoplasmic staining with a mixture of positively and negatively stained cells (+/-) at x400 magnification. Hematoxylin and eosin staining (H&E) staining to the right. (B) Immunohistochemical analysis of CACNA1H protein expression in case 19. PCC sample showed varied cytoplasmic staining (+/-) at x400 magnification. H&E staining to the right.
