# Supplementary material for: Genetic variants and down-regulation of CACNA1H in pheochromocytoma
Source: Endocr Relat Cancer. 2024 Jul 8;31(9):e230061. doi: 10.1530/ERC-23-0061 (PMC11301417; doi:10.1530/ERC-23-0061)
Supplement: Supplementary Figure S3. Enrichment analyses comparing CACNA1H variants to CACNA1H wild-type cases. Data from cbioportal/TCGA and analyses/graphs were made using WebGestalt. [file supplementary_figure_3.pdf]

A

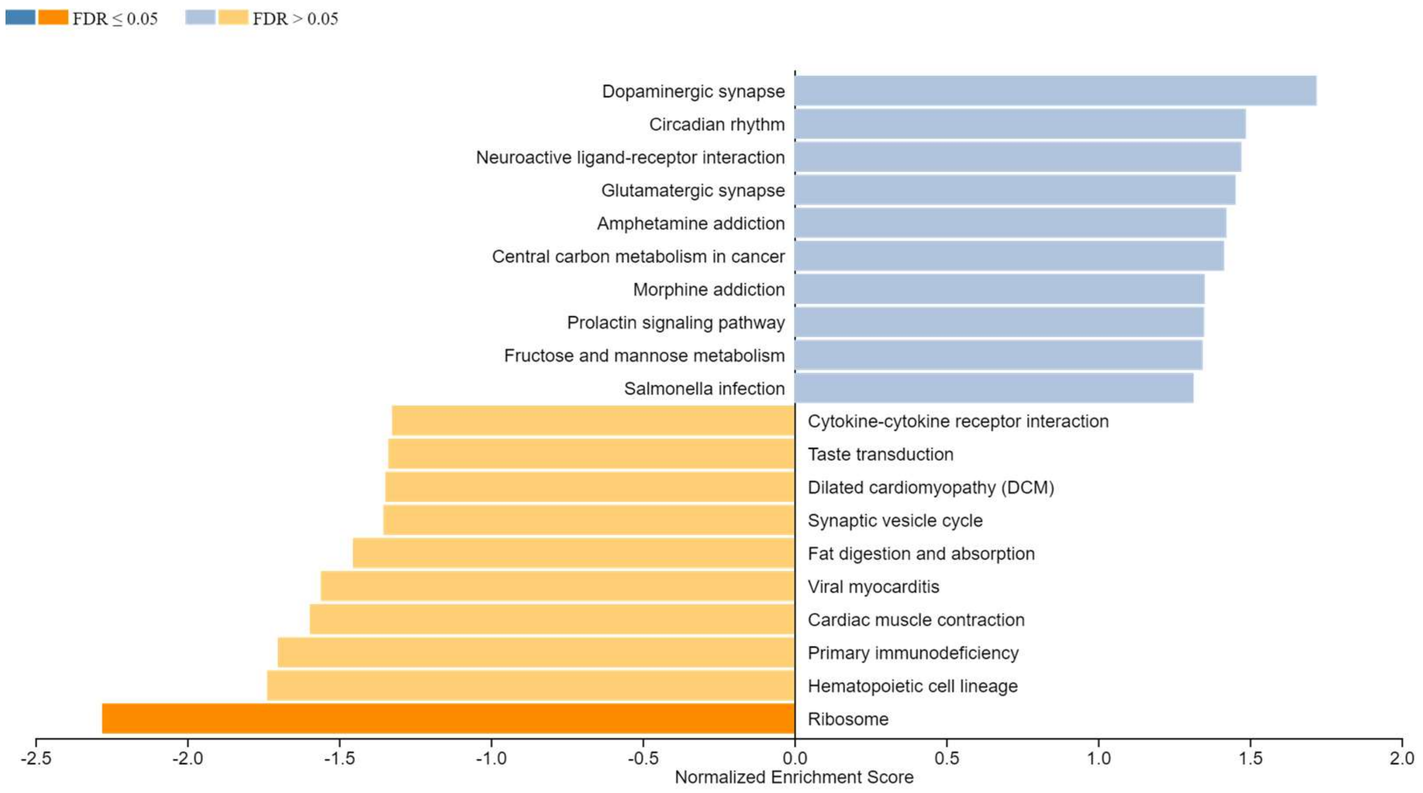

B

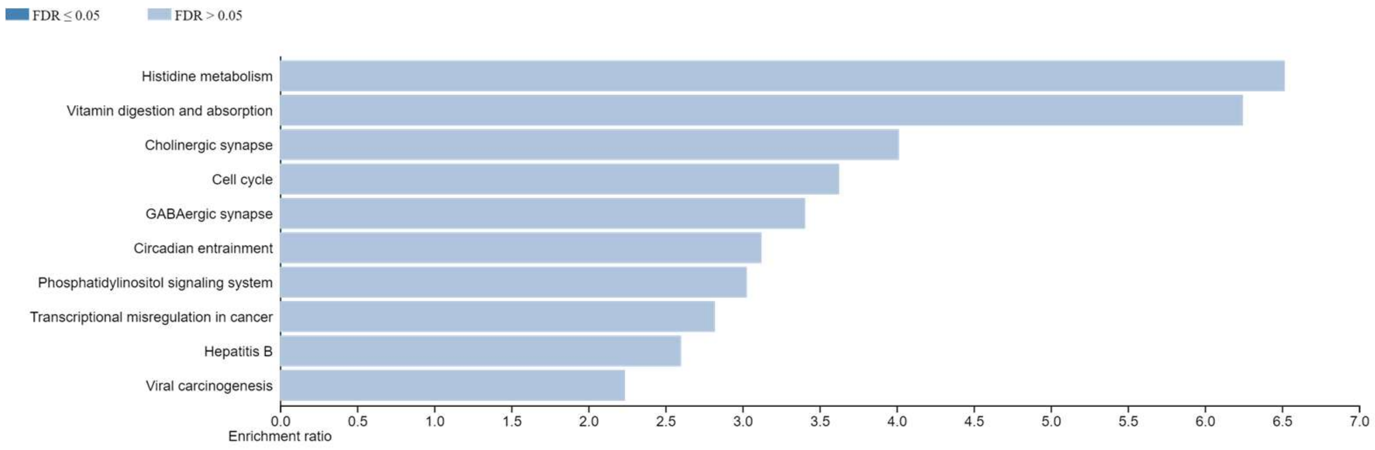

Supplementary Figure S3. Enrichment analyses comparing CACNA1H variants to CACNA1H wild-type cases. Data from cbioportal/TCGA and analyses/graphs were made using WebGestalt. A.) Gene Set Enrichment Analysis (GSEA) comparing 17338 available genes between the groups with a CACNA1H variant(n = 3) to the tumors with no CACNA1H alteration (n = 174). B.) Over representation analysis (ORA) comparing 278 significantly DEGs between the group with CACNA1H altered tumors (n = 3) and non-CACNA1H altered tumors(n = 174).
